# Altering gene expression by aminocoumarins: the role of DNA supercoiling in *Staphylococcus aureus*

**DOI:** 10.1186/1471-2164-15-291

**Published:** 2014-04-16

**Authors:** Wiebke Schröder, Jörg Bernhardt, Gabriella Marincola, Ludger Klein-Hitpass, Alexander Herbig, Guido Krupp, Kay Nieselt, Christiane Wolz

**Affiliations:** 1 Interfaculty Institute of Microbiology and Infection Medicine, University of Tübingen, Elfriede-Aulhorn-Strasse 6, 72076 Tübingen, Germany; 2 Institute for Microbiology, Ernst-Moritz-Arndt-University Greifswald, F.-L.-Jahn-Str. 15, Greifswald, Germany; 3 Institut für Zellbiologie, Universitätsklinikum Essen, Virchowstraße 173, 45122 Essen, Germany; 4 Centre for Bioinformatics Tübingen, University of Tübingen, Sand 14, 72076 Tübingen, Germany; 5 AmpTec GmbH, Königstraße 4A, 22767 Hamburg, Germany; 6 Present address: Research Centre for Infectious Diseases ZINF, University of Wuerzburg, Josef Schneider Str.2/ D15, Wuerzburg, Germany

**Keywords:** St*aphylococcus aureus*, Supercoiling, Gyrase, *arlR*, Spacer, Aminocoumarins, Novobiocin, Microarray, Voronoi tree map

## Abstract

**Background:**

It has been shown previously that aminocoumarin antibiotics such as novobiocin lead to immediate downregulation of *recA* expression and thereby inhibit the SOS response, mutation frequency and recombination capacity in *Staphylococcus aureus*. Aminocoumarins function by inhibiting the ATPase activity of DNA gyrase subunit B with a severe impact on DNA supercoiling.

**Results:**

Here, we have analysed the global impact of the DNA relaxing agent novobiocin on gene expression in *S. aureus*. Using a novobiocin-resistant mutant, it became evident that the change in *recA* expression is due to gyrase inhibition. Microarray analysis and northern blot hybridisation revealed that the expression levels of a distinct set of genes were increased (*e.g., recF-gyrB-gyrA*, the *rib* operon and the *ure* operon) or decreased (*e.g., arlRS, recA, lukA, hlgC* and *fnbA*) by novobiocin. The two-component ArlRS system was previously found to decrease the level of supercoiling in *S. aureus*. Thus, downregulation of *arlRS* might partially compensate for the relaxing effect of novobiocin. Global analysis and gene mapping of supercoiling-sensitive genes did not provide any indication that they are clustered in the genome. Promoter fusion assays confirmed that the responsiveness of a given gene is intrinsic to the promoter region but independent of the chromosomal location.

**Conclusions:**

The results indicate that the molecular properties of a given promoter, rather than the chromosomal topology, dictate the responsiveness to changes in supercoiling in the pathogen *Staphylococcus aureus*.

## Background

Bacterial chromosomes are usually composed of circular, double-stranded DNA in an overall negatively supercoiled state. Various conditions such as osmotic stress, oxygen tension, temperature changes, the growth phase and certain antibiotics (*e.g.,* aminocoumarins) can change the level of supercoiling, which in turn affects transcription, DNA replication and chromosomal segregation (for reviews, see [1-6]). Three enzymes are important for maintaining a steady-state level of supercoiling: topoisomerase I, gyrase and topoisomerase IV. Topoisomerase I introduces single-stranded DNA breaks and rotate the DNA of one single strand of the double-helix around the other. Thereby negative supercoils are removed. Gyrase is unique in introducing negative supercoils into DNA, whereas topoisomerase IV primary function is decatenation of DNA at the end of replication [7]. DNA gyrase is a tetramer with two identical subunits of GyrA and GyrB. GyrA subunit is involved in DNA breakage and religation. The N-terminal domain of GyrB subunits contains the ATPase active side [8]. DNA gyrase subunit B is the primary target of different aminocoumarin-based antibiotics [9], which competitively inhibit the ATPase activity, stabilising the DNA complex without inducing double-strand breaks [8]. Thereby, aminocoumarins (*e.g.,* novobiocin) specifically cause DNA relaxation in living bacteria, a property often used to study the impact of supercoiling on gene expression.

Thus far, the impact of supercoiling on gene transcription on a global scale has been analysed in *Escherichia coli*[10,11], *Haemophilus influenzae*[12], *Helicobacter pylori*[13] and *Streptococcus pneumoniae*[14]. Based on the results obtained in *E. coli*[11], it was proposed that nucleoid-associated proteins such as FIS (factor for inverse stimulation) or H-NS (histone-like nucleoid structuring protein) determine the spatial distribution of genes and their sensitivity to supercoiling. Of note, homologues of FIS and H-NS are lacking in Firmicutes such as *Staphylococci* or *Streptococci*, and much less is known about the role of supercoiling in these organisms. The results obtained in *S. pneumoniae* indicate that the genome of this organism is organised in large topology-reacting gene clusters that determine whether a gene is repressed or activated after exposure to the relaxing agent novobiocin [14].

The question of whether and how supercoiling influences gene expression in *S. aureus* has been rarely addressed [15,16]. In these studies, the impact of novobiocin treatment on the expression of selected virulence genes, such as *spa* (which encodes protein A) and *eta* (which encodes exfoliative toxin A), was analysed. The two-component regulatory system, ArlRS, was proposed to be involved in the regulation of supercoiling [15]. ArlRS regulates the expression of genes involved in different functions, including autolysis, cell division, growth and pathogenesis [17-19].

In a previous study, we compared the impact of two different gyrase inhibitors, ciprofloxacin and novobiocin, on the SOS response in *S. aureus*[20]. Ciprofloxacin is a prototypic quinolone, which, in contrast to aminocoumarins, interferes with the gyrase A subunit and induces double-strand breaks in DNA, thereby activating the SOS response. The double-strand breaks are processed to ssDNA, on which RecA forms filaments. The activated RecA complex induces autocleavage of the repressor LexA, which then allows for the transcription of genes involved in DNA repair, as well as *recA* and *lexA* themselves [21,22]. We showed that aminocoumarins inhibited the ciprofloxacin-mediated SOS response. This was due to severe inhibition of *recA* expression by aminocoumarins at the transcriptional level. The *recA* inhibition was presumably due to alterations in supercoiling and was LexA-independent.

Here, we show that inhibition of *recA* expression is tightly linked to inhibition of the gyrase B subunit. The imposed inhibition of gyrase B by aminocoumarins resulted in distinct alterations in gene expression. The supercoiling sensitivity was independent of the Arl system or the chromosomal location. Thus, in contrast to mechanisms proposed for other organism [11,14], supercoiling sensitivity in *S. aureus* is intrinsic to the promoter region of a given gene but autonomous from proposed topology-linked gene clusters [11].

## Results

### The effects of aminocoumarins on *recA* gene expression are due to GyrB inhibition

We previously showed that different aminocoumarins cause severe inhibition of *recA* transcription [20]. This effect is likely mediated by the known inhibition of the GyrB subunit. To verify this assumption, a strain expressing a non-susceptible GyrB enzyme was analysed. A mutated *gyrB* gene (Ile102Ser, Arg144Ile) [23,24] was transduced into strain HG001, resulting in strain HG001 *nov142*. This strain is resistant to novobiocin and clorobiocin but sensitive towards ciprofloxacin and simocylinone D8 (SD8), with MICs of 80 mg/l, 0.25 mg/l, and 4 mg/l for novobiocin, ciprofloxacin and SD8, respectively. SD8 is a hybrid antibiotic composed of an aminocoumarin and a polyketide element with a different mode of action. This antibiotic interacts with two separate pockets of the gyrase enzyme, situated in the GyrA and GyrB subunits [25].

**Figure 1:**
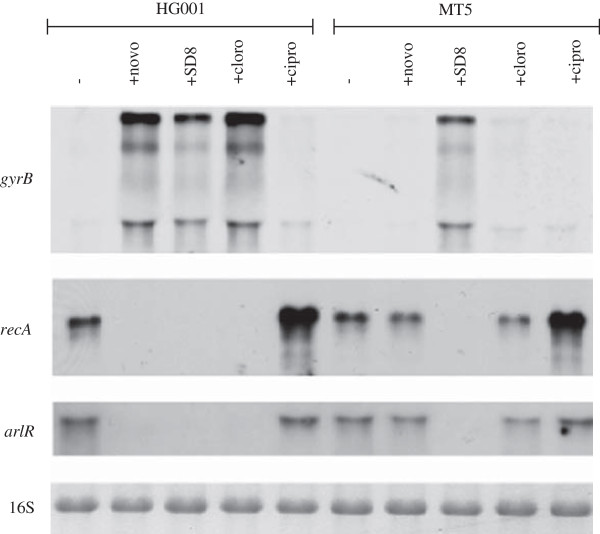
**Changes in gene expression by aminocoumarins is due to gyrase inhibition.** Strain HG001 and HG001, *nov142* were grown to exponential phase (OD_600_ = 0.6) and treated with novobiocin (novo, 0.5 mg/L), simocyclinone D8 (SD8, 4 mg/L), clorobiocin (cloro, 0.125 mg/L), and ciprofloxacin (cipro, 40 mg/L) for 10 min. RNA was hybridized with a digoxigenin-labelled *recA* as well as *gyrB* and *arlR* specific probes.

The parental strain responded to novobiocin, clorobiocin and SD8 by downregulation of *recA* (Figure 1). SD8 is active in strain HG001 *nov142* and resulted in the severe inhibition of *recA* transcription, comparable to that of the parental strain. In contrast, novobiocin and clorobiocin did not have any inhibitory effects on *recA* transcription in the resistant *gyrB* mutant HG001 *nov142* (Figure 1). This indicates that the effect on *recA* transcription is mediated by aminocoumarins-dependent GyrB inhibition and is unlikely to be due to additional effects on other potential targets. In agreement with the different mode of action and the induction of the SOS response, the quinolone ciprofloxacin resulted in up-regulation of *recA* independently on the *gyrB* mutation*.*

We further analysed the effects of aminocoumarins on *gyr* genes. Northern blot analysis using a *gyrB*-specific probe detected a transcript > 5 Kbp, which is consistent with a predicted operon structure composed of *recF*, *gyrB* and *gyrA* (Figure 1). Expression of this operon was not influenced by the addition of ciprofloxacin. In contrast, the aminocoumarins resulted in a severe induction of the *recF-gyrB-gyrA* operon. This induction was again mediated by GyrB inhibition because the *nov142* strain only responded to SD8 but not to novobiocin or clorobiocin (Figure 1). Notably, the change in gene expression was observed after only 10 min of antibiotic exposure.

It was proposed that the two-component regulatory system ArlRS is involved in the regulation of supercoiling and supercoiling-sensitive genes [15]. We therefore analysed the effect of the aminocoumarins on *arlRS* expression. This operon was found to be severely downregulated by novobiocin and clorobiocin in the parental strain but not in the *gyrB* mutant strain (Figure 1).

In conclusion, inhibition of Gyrase B by aminocoumarins has a distinct effect on the expression of different operons: the *recF-gyrB-gyrA* operon is upregulated, whereas the expression of *arlRS* and *recA* is inhibited. These effects are most likely due to alterations in the level of supercoiling due to inhibition of the GyrB ATPase activity.

### Global transcriptional response to novobiocin treatment

**Figure 2:**
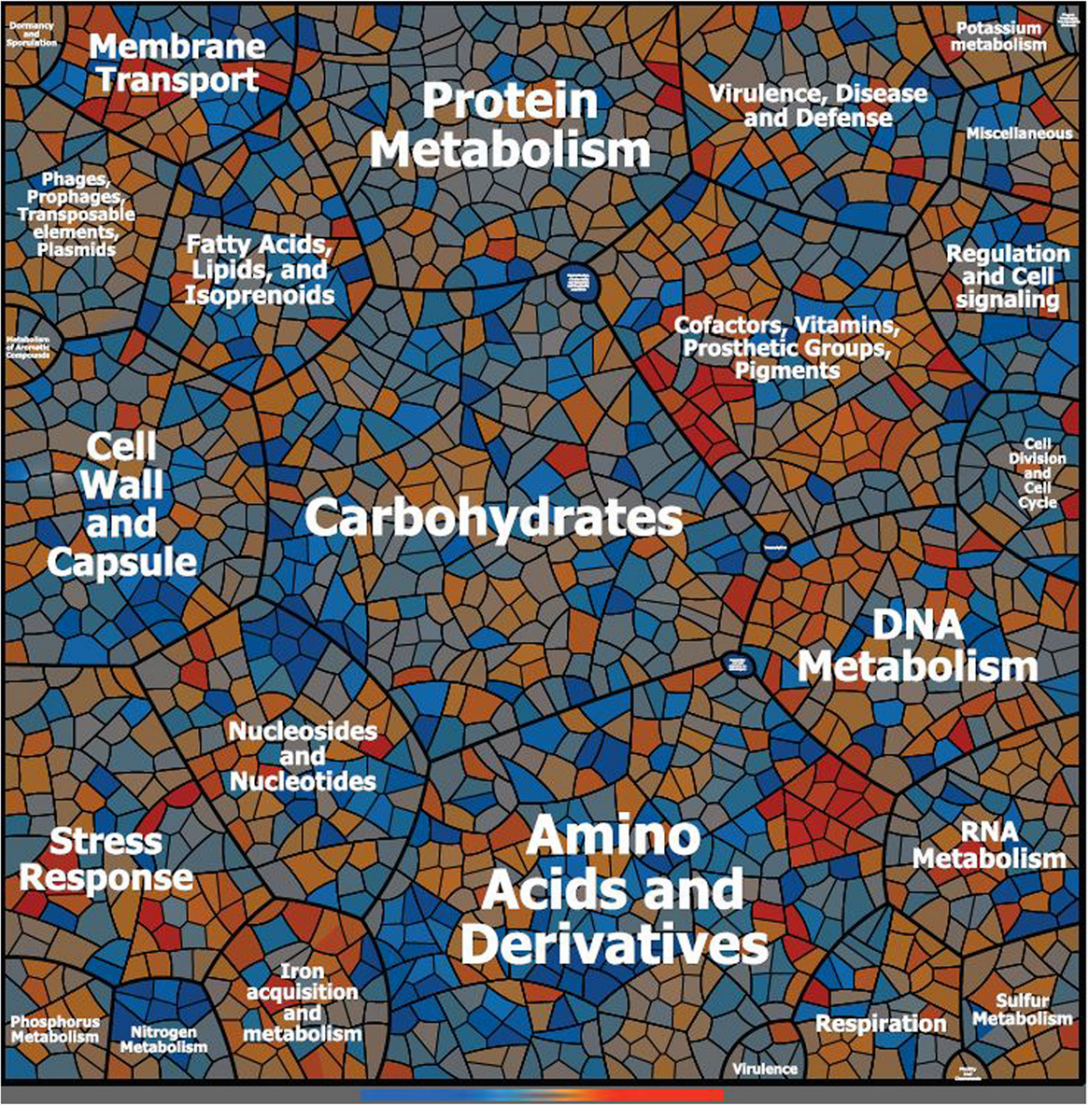
**Global changes in gene expression to novobiocin treatment.** Gene expression differences between novobiocin treated (0.5 mg/L) and untreated HG001 determined by microarray analysis and illustrated by Voronoi Treemap (sub-function categories). Strain HG001 was grown to exponential phase (OD_600_ = 0.6) and treated with and without novobiocin (0.5 mg/L) for one hour. Triplicate RNA samples were processed and further analyzed with Affymetrix gene chip. All functionally annotated genes of the *S. aureus* Affymetrix array are displayed and allocated to TheSeed functional categories. Clustering in groups or categories means functional or systematic relationship. If a gene or a group of genes fulfills multiple functions, it will be allocated to more than one functional group and by this reason appear at multiple locations within the treemap. Voronoi tree map is shown by a colour ramp from dark blue (at least 0.5 (log_2_fold) repressed in novobioin treated bacteria) via light grey (unchanged) to dark orange (at least 0.5 (log_2_fold) induced in novobiocin treated cells).

To obtain a more comprehensive overview on the effect of GyrB inhibition, and thus relaxation of supercoiling, we performed microarray analysis. Bacteria were grown to OD_600_ = 0.6 followed by one hour of incubation with or without novobiocin. Microarray analysis confirmed that *recA* and *arlRS* expression levels were significantly reduced in the treated samples, whereas the expression levels of *recF*, *gyrA* and *gyrB* were significantly increased (Additional file 1: Table S1). The data were visualised as a Voronoi treemap (Figure 2 and Additional file 2: Figure S1). Overall, 11% of the genes were influenced by treatment with novobiocin. A total of 166 genes were found to be significantly downregulated and 114 genes were upregulated by novobiocin. The largest inhibitory effect was observed for *lukA*, which encodes a bi-component leukotoxin subunit of LukAB. Other virulence genes that appeared to be downregulated by novobiocin were *hlgC (*Gamma-hemolysin*), sak* (staphylokinase*), fnb* (fibronectin-binding protein A/B) and the *cap* operon (coding for biosynthesis genes required for the synthesis of capsular polysaccharides) (Figure 2). On the other hand, aside from the *recF-gyrB-gyrA* operon, metabolic operons coding for enzymes involved in riboflavin biosynthesis (*rib*A-H), iron transport (*fhuA-G*) and urease (*ure*A-G) were activated under novobiocin treatment.

### ArlR does not mediate the supercoiling effect on virulence genes

**Figure 3:**
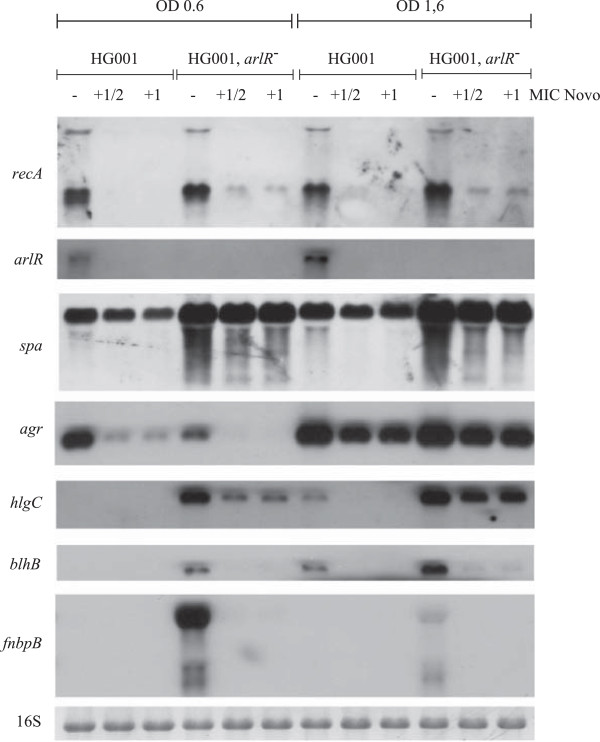
**Influence of novobiocin on ArlR target genes.** Strain HG001 and HG001, *arlR*^*-*^ were grown to exponential phase (OD_600_ = 0.6) as well as later exponential phase (+45 min = OD_600_ 1.6) and treated with and without novobiocin (Novo, 0.25 mg/L and 0.5 mg/L) for 5 min. RNA was hybridized with a digoxigenin-labelled *recA* as well as *gyrB, spa, hlgC, blhB, ribD, hrcA* and *arlR* specific probe.

The effect of novobiocin might be indirect and mediated by regulatory systems. In this regard, the *arlRS* system was of special interest [15]. For further analysis, an *arlR* mutant of strain HG001 was generated, and the effect of novobiocin on selected target genes was assessed. Because most of the genes of interest were strongly influenced by the growth phase, we added novobiocin at the mid-exponential (OD_600_ = 0.6) or post-exponential growth phase (OD_600_ = 1.6) (Figure 3). The *arlRS* operon was severely downregulated in both growth phases after the addition of novobiocin. In agreement with previous results from Liang *et al.,*[18] expression of RNAIII of the *agr* operon was diminished in the *arl* mutant at OD 0.6, whereas the virulence genes *lukAB*, *hlgC* and *fnbB* were upregulated at OD 0.6 and OD1.6 (Figure 3). However, the expression of the virulence genes (*lukAB, hlgC* and *fnbB*) was similarly inhibited by novobiocin in the wild type and the *arlR* mutant. Thus, although *arlRS* expression is highly sensitive to novobiocin, this effect is additive and independent of the effect of novobiocin on target gene expression (Figure 3).

We also analysed the expression of *spa* because it was described to be inhibited by ArlRS [19,26] but activated by novobiocin [15]. We were able to confirm that *spa* expression is elevated in an *arl* mutant when compared with the wild type (Figure 3). However, *spa* transcription was not found to be significantly affected and showed only slightly decreased expression under novobiocin treatment, similar to results obtained by others [27].

### Supercoiling-sensitive genes are not clustered in the *S. aureus* genome

**Figure 4:**
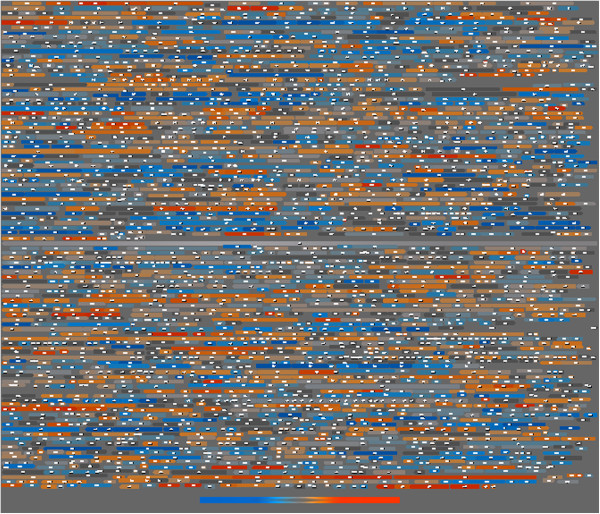
**Genome map of *S. aureus *HG001 genes, differently expressed after novobiocin treatment.** Colour ramp from dark blue (at least twofold repressed in novobiocin treated bacteria) via light grey (unchanged) to dark orange (at least twofold induced in novobiocin treated cells). Strain HG001 was grown to exponential phase (OD_600_ = 0.6) and treated with and without novobiocin (0.5 mg/L) for one hour.

For *S. pneumoniae* it was described that supercoiling-sensitive genes are organised in clusters [14]. To analyse the distribution of genes affected by supercoiling, we mapped the novobiocin-responsive genes on the chromosome of *S. aureus*. As illustrated in Figure 4, novobiocin-responsive genes are randomly distributed throughout the genome. Genes appearing clustered are those co-transcribed in one operon.

### Supercoiling sensitivity is intrinsic to the promoter elements

**Figure 5:**
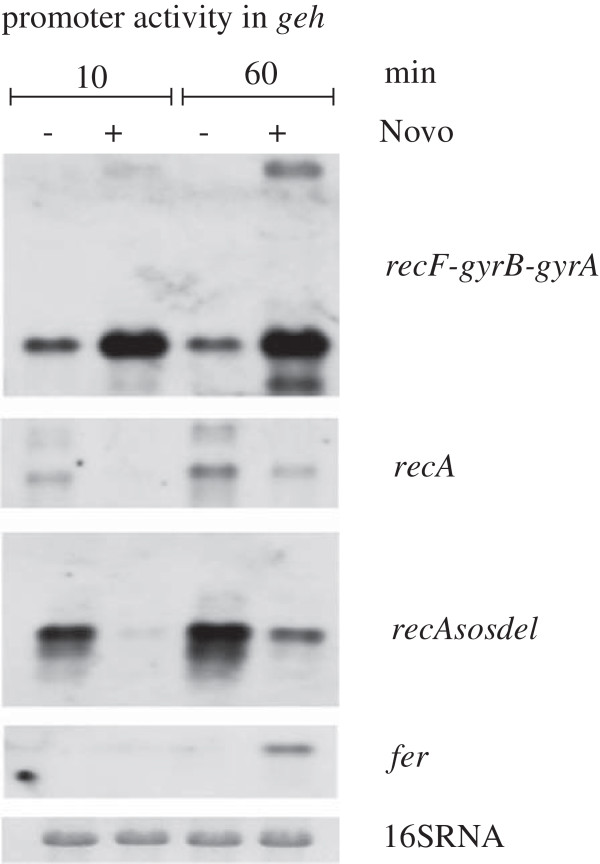
**Changing the position of promoters in *S. aureus *genome does not play a role in responsiveness to novobiocin.** Strain HG001 harboring integration plasmids pCG350, pCG351, pCG352 and pCG357 were grown to exponential phase (OD_600_ = 0.6) and further incubated with and without novobiocin (Novo, 0.5 mg/L) for 10 and 60 min. The promoter regions of the indicated genes were cloned in front of a truncated *gfp* gen and expression monitored by hybridization with a digoxigenin-labelled *gfp* probe. *Recdelsol* indicates that the LexA binding motif was deleted from the *recA* promoter region.

To further analyse whether the responsiveness of supercoiling-affected genes is dependent on their chromosomal location, the promoters of selected supercoiling-responsive genes were dislocated from their native environment. Specifically, these promoters were placed into the lipase gene *geh*, *w*hich appeared to be downregulated upon novobiocin treatment (Figure 5). The promoters were cloned in front of a truncated *gfpmut3.1* gene (lacking the RBS and start site), and all plasmids were inserted into *geh*. The expression of the truncated *gfp* mRNA was used as marker for the activity of the subcloned promoters after treatment with novobiocin for 10 and 60 min (Figure 5). All tested promoters retained their original responsiveness towards novobiocin: the promoters from the *recF-gyrA-gyrB* operon and from *fer* were upregulated by novobiocin at its native location, and these promoters also showed an increase in activity when they were located in *geh*. In contrast, the *recA* promoter was inhibited at its native location (Figure 1) and when localised in *geh* (Figure 5). Thus, the localisation on the chromosome does not dictate the response to supercoiling.

RecA is a prototypic LexA target gene, and one could assume that the supercoiling sensitivity of the promoter occurs through interference of local changes in DNA topology with LexA. We therefore deleted the LexA binding domain from the *recA* promoter region. As expected, the deletion of the *lexA-*binding motif resulted in increased, LexA-independent promoter activity. However, the truncated promoter element was still novobiocin sensitive (Figure 5).

## Discussion

Here, we determined the transcriptional response to the DNA relaxing agent novobiocin in *S. aureus*. Only a distinctive set of operons were found to be sensitive to supercoiling. In total, 11% of the genes were influenced by novobiocin. This is in good agreement with results observed in *E. coli*, in which 7% of the genome was affected [10].

We were able to show that *recA* transcription in *S. aureus* was sensitive to novobiocin treatment. This indicates that the *recA* promoter is highly dependent on DNA supercoiling imposed by active gyrase. Novobiocin had no impact on *recA* transcription in a strain with a mutation in *gyrB* (*nov142*). Thus secondary effects of novobiocin on other potential targets do not play a role in this regard. Of note, the novobiocin effect was independent of LexA because a similar effect of novobiocin on *recA* transcription was observed in an artificial *recA* promoter lacking the LexA binding motif. Thus, *S. aureus* is able to sense supercoiling to modulate the SOS response by adjusting the RecA level in the cell. In this way, aminocoumarins can counteract SOS-inducing conditions and their consequences, such as those imposed by ciprofloxacin [20].

In *S. aureus*, the *gyrA and gyrB* subunits are co-transcribed with *recF*, and the whole operon was severely upregulated after novobiocin treatment. *GyrB* is widely used as reference gene in qRT-PCR because it was shown that the expression of *gyrB* (and/or *recF*) is not influenced by major virulence regulators or different growth conditions [28]. In addition, the expression of the operon was also found to be insensitive towards ciprofloxacin (Figure 1). In many other organisms, including *S. pneumoniae* and *E. coli*, *gyrA* and *gyrB* are distantly located, and the expression of these genes is independently regulated by several factors, including nucleoid-associated proteins. In *S. aureus*, the promoter preceding *recF-gyrB-gyrA* has presumably evolved to directly measure supercoiling imbalance, leading to upregulation of gyrase under relaxed conditions. Thus far, the environmental conditions that can impose such changes in *S. aureus* remain unclear.

Microarray analysis further reveals that several additional genes are influenced by novobiocin treatment. Some of these genes are presumably indirectly affected through secondary regulatory mechanisms. In this regard, the profound inhibition of the *arlRS* operon by novobiocin was of special interest. By searching microarray databases [29], this operon was not described to be differentially expressed by other regulatory mechanisms in *S. aureus*, indicating that the *arlRS* promoter itself is sensitive to supercoiling. Interestingly, the *arlRS* system was previously described to be involved in regulating the supercoiling level in *S. aureus*. Deletion of *arlRS* resulted in an increased level of supercoiling, an effect opposite to that of novobiocin treatment [15]. Thus, downregulation of *arlRS* under novobiocin treatment can be viewed as a compensatory mechanism. In agreement with this assumption is the observation that, for some virulence factors, the impact of *arlRS* mutation is opposite to that of novobiocin treatment, and several of the genes described to be under the control of *arlRS*[18] were also found to be influenced by novobiocin. However, analysis of a selected set of genes showed that the effect of novobiocin observed in the *arl* mutant strain (Figure 3) is similar to that in the wild type, indicating that novobiocin affects gene transcription independent of *arlRS*.

It is well recognised that the level of supercoiling is highly dynamic and affects gene expression directly and distinctly. However, the reason why different genes have different sensitivities towards supercoiling is under debate. In *E. coli*, a crosstalk between DNA supercoiling and nucleoid-associated proteins is involved in coordinated gene expression. The spatial ordering of genes along the chromosome corresponds to an inferred gradient of superhelical density [30,31]. *S. pneumoniae*, like *S. aureus*, lacks many of the major nucleoid-associated proteins. In this organism, genes responding to changes in the level of supercoiling were found to be organised in chromosomal clusters. According to these topology-related models, the localisation of a given gene would dictate whether it is positively or negatively regulated by changes in supercoiling. In our analysis, we were unable to confirm such an association by mapping the responsive genes along the chromosome (Figure 4). In our analysis 11% of genes of *S. aureus* responded to novobiocin. By choosing different concentration of the drug and incubation time, the amount of responsive genes may vary and might impact the outcome of cluster analysis. Also apart from supercoiling, the folded nucleoid also forms tertiary structures, which might form clustered regions which might be missed using a simple mapping of genes along the chromosome.

Nevertheless, dislocation of three different promoters showed that the responsiveness of a given gene is determined by the promoter region and probably independent of the chromosomal localisation. These results are similar to analyses of the *E. coli gyrA* and *gyrB* promoters, both of which are activated under relaxing conditions [32]: a reporter gene fused with *gyrA* or *gyrB* sequence was inducible by an aminocoumarin, also suggesting that only a small region of DNA is necessary for supercoiling sensitivity. In *E. coli*[10] and *Streptococcus pneumonia* supercoiling sensitive genes were characterized by a different composition of nucleotides, with a higher AT content in upregulated genes. However, in *S. aureus* the AT content of up versus downregulated promoters were found not to differ (unpublished observation).

Our findings may be in line with a previous assumption that the supercoiling responsiveness of a gene may be correlated with the length of the spacer region between the -35 and -10 regions [33,34]. According to this model, expression should be higher at a low level of supercoiling for genes with short spacers (less than the optimal 17 bp) and higher at elevated levels of supercoiling for genes with long spacers (greater than 17 bp). Spacing was shown to influence supercoiling sensitivity in *E. coli*[35] and *Helicobacter pylori*[13]. Our results also indicate that the length of the spacer might play a role in the supercoiling sensitivity of a given gene. For example, the highly upregulated gene *gyrA* has a relative short spacer, whereas the down regulated gene *recA* has a longer spacer.

## Conclusion

Most effects of novobiocin treatment, such as a decrease in *recA* or increase in *recF-gyrB-gyrA*, are directly linked to a change in the level of supercoiling. Genes that are sensitive to supercoiling are not clustered on the chromosome of *S. aureus*, and responsiveness is also independent of the chromosomal location. The relationship between promoter spacing and gene regulation in response to changing levels of supercoiling will require further investigation. Additionally, the physiological conditions leading to different supercoiling states in *S. aureus* are so far not thoroughly explored.

## Methods

### Strains and growth conditions

The strains and plasmids used in this study are listed in Additional file 3: Table S2. The *S. aureus* strains were grown in tryptic soy broth (TSB media; Oxoid, Hampshire, United Kingdom). For the strains carrying resistance genes, antibiotics were only used in overnight cultures at the following concentrations: tetracycline (5 mg/L) and chloramphenicol (10 mg/L). Bacteria from an overnight culture were diluted to an initial optical density (OD) 600 nm (OD_600_) of 0.05 in fresh medium and grown with shaking (220 rpm) at 37°C to the desired growth phase. Antibiotics were added as indicated related to the minimal inhibitory concentration (MIC) of the strains determined as previously described [20]. SD8 was a gift from H.P. Fiedler (University Tübingen, Insitut für Mikrobiolgie/Biotechnologie, Tübingen, Germany) and clorobiocin was donated by L. Heide (University Tübingen, Insitut für Pharmazie, Tübingen, Germany).

### Construction of mutants

Transduction using Φ11 lysates of strain MT5 [23] and SM99 [36] created HG001 *nov142* and HG001 *arlR*^*-*^ respectively.

### RNA isolation and northern blot analysis

RNA was isolated as previously described [28]. Briefly, bacteria were lysed in 1 ml of Trizol reagent (Invitrogen, Darmstadt, Germany) with 0.5 ml of zirconia-silica beads (0.1 mm diameter, Carl Roth, Karlsruhe) in a high-speed homogenizer (Savant Instruments, Farmingdale, NY, USA) and the RNA was purified as described in the instructions provided by Invitrogen. Northern analyses were performed as described by Goerke *et al.*[28], using 2 μg of RNA per lane. Digoxigenin (DIG)-labeled probes for the detection of *recA* transcripts were generated using a DIG-labeling PCR kit, as described by the manufacturer (Roche Biochemicals, Mannheim, Germany) and the oligonucleotides listed in supplemental Additional file 3: Table S2.

### Promoter activity assay

Promoter of interest were cloned in front of the promoterless *gfpmut3.1* gene in plasmid pCG188 [37] and integrated into the lipase gene *geh* in the chromosome of the analyzed strain. This allows for quantifying the expression of *gfpmut3.1*, detected by northern blotting, as equivalents of promoter activity.

A 14 bp deletion in the SOS box of the *recA* promoter was achieved by amplifying two PCR products with primer sosdel1for and sosdelinsrev for product A and sosdelinsfor and sosdel2rev for product B. By using primer EcoRIsosdelfor and EcoRIsosdelrev and product A and B as templates the promoter fragment recAsosdel was generated and subcloned into pCR4blunt-topo, for convenient usage.

Promoter fragments of *recF-gyrA-gyrB*, *recA*, *recAsosdel*, and *fer* were amplified by employing the oligonucleotides listed in supplemental Additional file 3: Table S2. The amplicons were ligated into the EcoR*I*-digested integration vector pCG188 [38] in front of the promoterless *gfpmut3.1* gene, yielding plasmid pCG352, pCG351, pCG350 and pCG357. The plasmids were verified by restriction digestion and PCR. The plasmids were transferred into competent *S. aureus* CYL316 [38] and then transduced into *S. aureus* HG001. RNA was isolated, and detected by northern blot with a *gfp* specific probe.

### Microarray analysis

Cell pellets were lysed using mechanical disruption in a high-speed homogenizer (Savant Instruments, Farmingdale, NY, USA) with 0.5 ml zirconia beads (0.1 mm in diameter, Carl Roth, Karlsruhe) for 10 sec at a speed of 6,500 rpm in the presence of Trizol (Invitrogen, Darmstadt, Germany). The lysates were processed as described in the instructions of the RNA isolation kit ExpressArt RNAready (#9007-A100, AmpTec, Hamburg, Germany), including an on-column DNase treatment to remove genomic DNA.

Total RNA samples (2 μg) were amplified and biotin-labelled, using the ExpressArt Bacterial mRNA amplification Micro kit: selective amplification technology results in mRNA-enrichment and rRNA depletion. Fragmentation of biotinylated cRNA, hybridization, washing and staining of the arrays was done according to the standard Affymetrix GeneChip protocol (version2) on the GC Scanner 3000 with G7 update. 5 μg of biotin-labelled cRNA were hybridized to GeneChip *S. aureus* Genome Array at the BioChip-Labor, Universitätsklinikum Essen. The *S. aureus* Affymetrix GeneChips used in this study are the most comprehensive commercially available arrays, representing genomic sequences from *S. aureus* strains, COL, N315, and Mu50, as well as intergenic regions. The experiment for each response was repeated three times (biological replicates). ANOVA statistical analysis was used to generate Additional file 1: Table S1. A multiple testing correction was applied based on corrected p-values (<0.05), generated by the step up method. Log Fold change was calculated and the threshold was set to 1. The data from microarray analysis have been deposited in NCBI’s Gene Expression Omnibus (GEO) and is accessible through GEO Series accession number GSE50157.

### Mapping microarray results on strain NCTC8325

The microarray raw data was normalized and summarized using robust multi-array analysis [39], which is integrated in the transcriptome analysis software Mayday-Seasight [40]. The microarray gene identifiers were mapped to *S. aureus* NCTC8325 gene locus tags by performing a BLAST alignment [41] of all ORF consensus sequences that were used as targets on the microarray to all annotated ORFs of *S. aureus* NCTC8325. To each microarray probe set, we assigned the locus tag of the best hit. However, if the sequence identity of the best hit was below 90%, the element was not included into the analysis.

### Voronoi tree map

Voronoi treemaps were constructed based on the data obtained from the microarrays [42,43] and the mapped genes of strain NCTC8325. All functionally annotated genes of the *S. aureus* Affymetrix array are displayed and allocated to TheSeed functional categories. Clustering in groups or categories means functional or systematic relationship. TheSeed is an open annotation repository continuously allocating genes of known and often still unknown function to functional classes according to the available scientific knowledge [43]. By this reason, some functional groups currently in curation may remain in a temporarily still unfinished state and therefore mostly labelled with abbreviations or explicit labels according to TheSeed standards. If a gene or a group of genes fulfils multiple functions, it will be allocated to more than one functional group and by this reason appear at multiple locations within the treemap. Gene expression differences between novobiocin treated and untreated samples have been encoded by a colour ramp from dark blue, (at least 0.5 (log_2_fold) repressed in novobioin treated bacteria) via light grey (unchanged) to dark orange (at least 0.5 (log_2_fold) induced in novobiocin treated cells).

### Map of supercoiled genes

In the genome of NCTC8325 starts and ends of genes were extracted, and gene arrows inserted. Each was colored by its specific colour according to the Voronoi Tree map.

## Competing interests

JB works with DECODON GmbH Greifswald. DECODON sells PAVER, a software for functional genomics data visualization and analyses which uses alogirthms for the generation of the shown treemap based data visualizations. The other authors declare that they have no competing interests.

## Authors’ contributions

WS and CW planned the experiments and wrote the manuscript. WS performed the experimental work. GM helped in cloning the *gfp* promoter constructs. GK amplified and labeled the RNA for Microarray analysis. LK performed the Microarray as well as the analysis of row data. AH and KN mapped Microarray results on strain 8325. JB designed and analyzed the Voronoi Tree Maps. CW conceived and supervised the project. All authors read and approved the final manuscript.

## Supplementary Material

Additional file 1: Table S1 Results from microarray analysis (+/- novobiocin). Click here for file

Additional file 2: Figure S1 Gene expression differences between novobiocin treated (0.5 mg/L) and untreated HG001 determined by microarray analysis and illustrated by Voronoi Treemap shown by a colour ramp from dark blue (at least 0.5 (log_2_fold) repressed in novobioin treated bacteria) via light grey (unchanged) to dark orange (at least 0.5 (log_2_fold) induced in novobiocin treated cells). Loci IDs refer to the Oklahoma 8325 *S. aureus* genome sequence (SAOUHSC). Click here for file

Additional file 3: Table S2 Strains/plasmids and oligonucleotides used in this work. Click here for file
