# Supplementary figures and images for: Altering gene expression by aminocoumarins: the role of DNA supercoiling in Staphylococcus aureus
Source: BMC Genomics. 2014 Apr 16;15:291. doi: 10.1186/1471-2164-15-291 (PMC4023603; doi:10.1186/1471-2164-15-291)

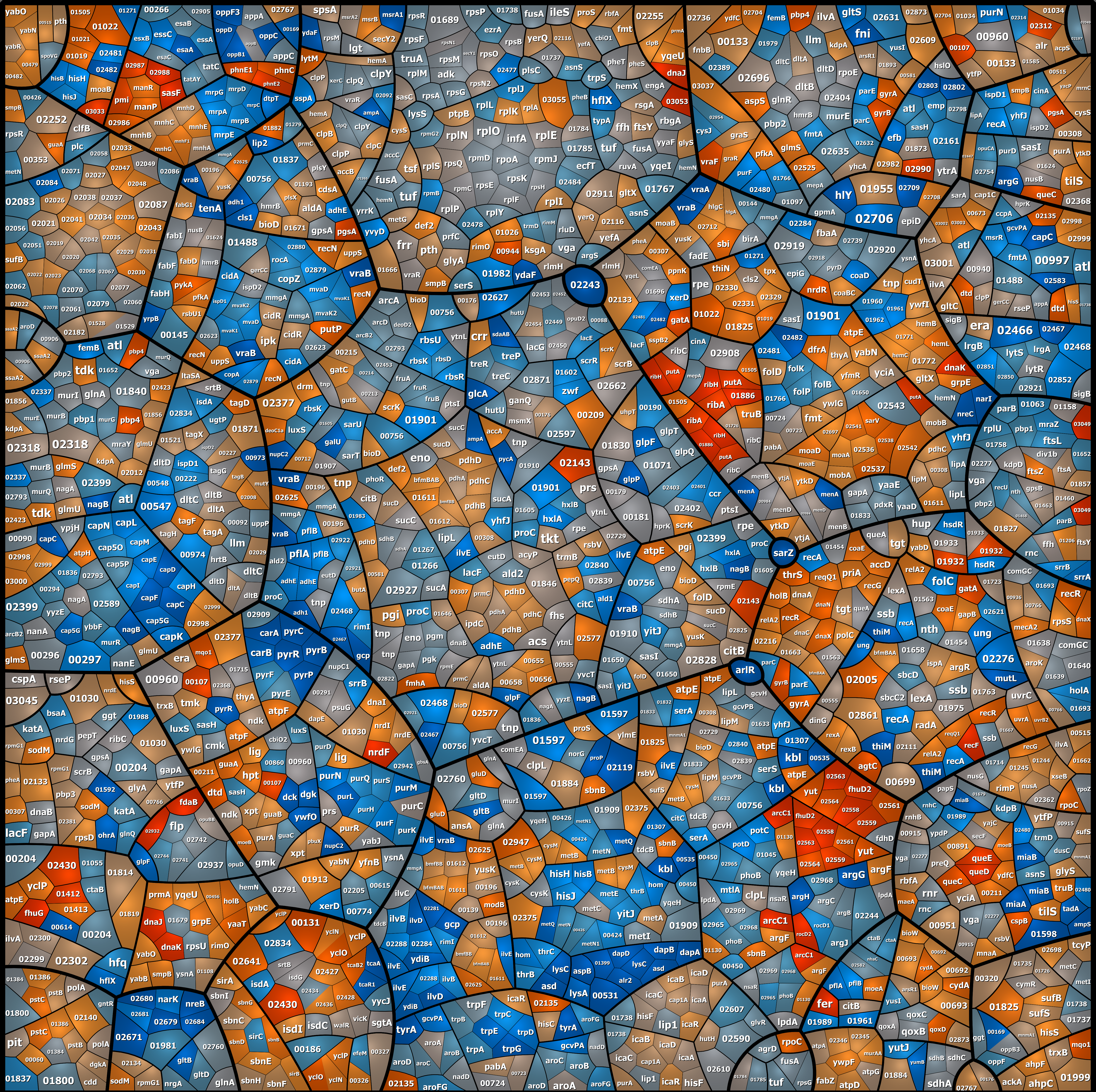

Supplement: Additional file 2: Figure S1 — Gene expression differences between novobiocin treated (0.5 mg/L) and untreated HG001 determined by microarray analysis and illustrated by Voronoi Treemap shown by a colour ramp from dark blue (at least 0.5 (log2fold) repressed in novobioin treated bacteria) via light grey (unchanged) to dark orange (at least 0.5 (log2fold) induced in novobiocin treated cells). Loci IDs refer to the Oklahoma 8325 S. aureus genome sequence (SAOUHSC). [file 1471-2164-15-291-S2.pdf]
